# Conical Tomography of a Ribbon Synapse: Structural Evidence for Vesicle Fusion

**DOI:** 10.1371/journal.pone.0016944

**Published:** 2011-03-01

**Authors:** Guido A. Zampighi, Cataldo Schietroma, Lorenzo M. Zampighi, Michael Woodruff, Ernest M. Wright, Nicholas C. Brecha

**Affiliations:** 1 Department of Neurobiology, David Geffen School of Medicine at UCLA, University of California Los Angeles, Los Angeles, California, United States of America; 2 Department of Physiology, David Geffen School of Medicine at UCLA, University of California Los Angeles, Los Angeles, California, United States of America; 3 Integrative Biology and Physiology, College of Letters and Science, University of California Los Angeles, Los Angeles, California, United States of America; 4 Department of Medicine, David Geffen School of Medicine at UCLA, University of California Los Angeles, Los Angeles, California, United States of America; 5 Jules Stein Eye Institute, David Geffen School of Medicine at UCLA, University of California Los Angeles, Los Angeles, California, United States of America; 6 Veterans Administration Greater Los Angeles Healthcare System, Los Angeles, California, United States of America; Dalhousie University, Canada

## Abstract

To characterize the sites of synaptic vesicle fusion in photoreceptors, we evaluated the three-dimensional structure of rod spherules from mice exposed to steady bright light or dark-adapted for periods ranging from 3 to 180 minutes using conical electron tomography. Conical tilt series from mice retinas were reconstructed using the weighted back projection algorithm, refined by projection matching and analyzed using semiautomatic density segmentation. In the light, rod spherules contained ∼470 vesicles that were hemi-fused and ∼187 vesicles that were fully fused (omega figures) with the plasma membrane. Active zones, defined by the presence of fully fused vesicles, extended along the *entire* area of contact between the rod spherule and the horizontal cell ending, and included the base of the ribbon, the slope of the synaptic ridge and ribbon-free regions apposed to horizontal cell axonal endings. There were transient changes of the rod spherules during dark adaptation. At early periods in the dark (3–15 minutes), there was a) an increase in the number of fully fused synaptic vesicles, b) a decrease in rod spherule volume, and c) an increase in the surface area of the contact between the rod spherule and horizontal cell endings. These changes partially compensate for the increase in the rod spherule plasma membrane following vesicle fusion. After 30 minutes of dark-adaptation, the rod spherules returned to dimensions similar to those measured in the light. These findings show that vesicle fusion occurs at both ribbon-associated and ribbon-free regions, and that transient changes in rod spherules and horizontal cell endings occur shortly after dark onset.

## Introduction

There is a remarkable diversity in the structure and protein composition of specialized regions of the plasma membrane, called “active zones,” where synaptic vesicles dock, fuse and release their transmitter [1]–[5]. Structurally, the active zone at conventional synapses in the central and peripheral nervous system is characterized by an electron-dense synaptic projection consisting of a web of particles, a corona of synaptic vesicles, a cytomatrix of filaments, proteins that regulate exocytosis and endocytosis, and synaptic vesicles that are hemi-fused (e.g., docked) or fully fused (e.g., forming an omega figure) with the plasma membrane [2], [6]–[9]. There is also a close alignment of the active zone with a postsynaptic specialization, known as the postsynaptic density [5], [10].

The active zone of photoreceptors, auditory and vestibular hair cells, and electroreceptors differ from conventional synapses. They contain at least one large and distinct electron-dense structure, which is shaped as a plate or sphere, called a synaptic ribbon or body [11]–[16]. Synaptic vesicles are distributed throughout the cytoplasm of the terminal and a small population of these vesicles is tethered to the synaptic ribbon. Synaptic vesicle fusion is thought to mainly occur at the base and in the immediate vicinity of the synaptic ribbon [17]–[22]. However, synaptic vesicles are also located and fuse at ribbon-free sites of saccular inner hair cells and goldfish bipolar cells [12], [23]–[26], implying a more extensive active zone in these cell types. There is variability in the plasma membrane of the active zone between the different sensory cell types. A plasma membrane thickening is present adjacent to the synaptic body of saccular inner hair cells [12], but not at the plasma membrane at the base of the synaptic ribbon or in ribbon-free regions of photoreceptor terminals [11], [13]–[15]. There is also a variable alignment of postsynaptic membrane densities and receptors; for instance, cochlear inner hair cell synaptic bodies are closely aligned with VIIIth nerve afferents [27]. In contrast, cone synaptic ribbons are distributed at variable distances to nearby horizontal cell endings, and more distant OFF-bipolar cell dendrites [13], [28], [29].

The site of transmitter release from photoreceptors has not been completely established. In both rods and cones, transmitter release is thought to mainly occur at an active zone located at the base of the synaptic ribbon where L-type Ca^2+^ channels are concentrated, and not from ribbon-free regions [11], [20], [30]–[34]. In addition, freeze-fracture studies of rabbit and monkey cones show fused synaptic vesicles along the base of the synaptic ribbon and the adjacent plasma membrane, referred to as the slope of the synaptic ridge [14]. In contrast to inner hair cells [12], [17], [23] there has been no evaluation of photoreceptor terminals focused on the distribution and location of hemi-fused and fully fused synaptic vesicles to map transmitter release sites in these cells.

The photoreceptor synapse releases glutamate continuously at a high rate in darkness [20], [35]–[37] and it is therefore ideally suited for *in vivo* studies concerning how physiological stimuli influence synaptic structure and transmitter release. We evaluated changes in rod spherule structure in light- and dark-adapted mice using conical electron tomography and density segmentation methods [9], [38]–[43]. This experimental approach addresses limitations caused by projecting the entire thickness of the section onto a single plane (“projection artifact”), which has severely limited studies using conventional electron microscopy due to the absence of depth information. The rod spherule reconstructions used in this study exhibit an isotropy in plane resolution of ∼3 or ∼6 nm depending on magnification, which is sufficient to identify hemi-fused and fully fused vesicles at the plasma membrane [43].

In retinas of both light- and dark-adapted mice, hemi-fused and fully fused vesicles were located along the entire surface of the rod spherule adjacent to horizontal cell axonal endings. The first 15 minutes of dark adaptation showed an increase of fully fused vesicles at ribbon-associated and ribbon-free regions, together with a decrease in the rod spherule volume and a concomitant increase in the surface area of the synaptic contact. These findings show for the first time that transmitter release occurs not only at the ribbon's base, but also at regions of the rod spherule located away from the ribbon.

## Results

### Rod Spherule

**Figure 1:**
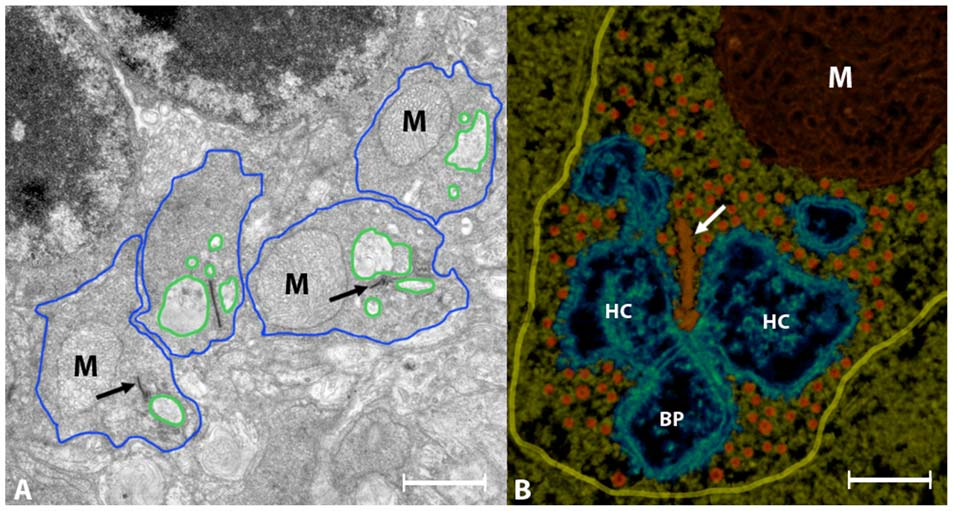
Rod photoreceptor axons of mouse retina. Panel A shows cross-sections of four rod spherules (blue lines). The principal organelles in the cytoplasm are electron-dense structure representing the synaptic ribbon (arrows), the mitochondria (M), and invaginations containing the endings of horizontal and bipolar cell processes (green lines). Synaptic vesicles are not resolved clearly at this magnification. Panel B shows a reconstructed ribbon synapse. The ribbon appears as a pole that is sandwiched between the endings of horizontal cell (HC) endings at each side and the bipolar cell (BP) dendrite is below the ribbon. The arciform density appears as a separate particle that links the base of the ribbon to the plasma membrane. Synaptic vesicles appear as spheres (orange) in the cytoplasm. The yellow line outlines the boundary of the axon terminal. Bars: A = 1.5 µm, B = 0.15 µm.

**Figure 2:**
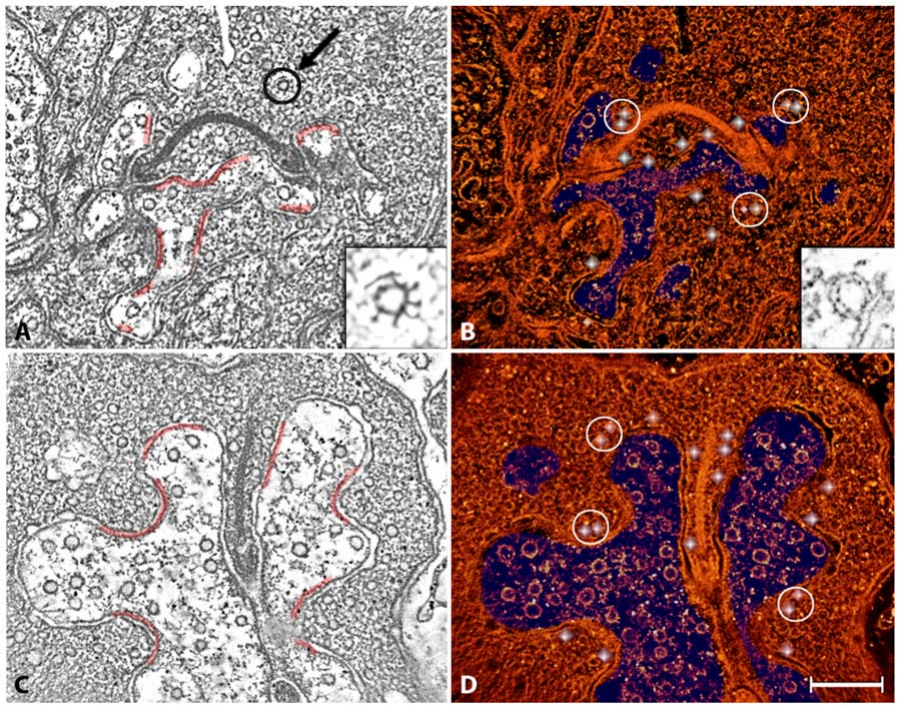
Distribution of docked vesicles at the region of synaptic contact. The figure shows orthogonal views of a rod synapse. Panel A shows a single plane of a reconstruction where the synapse is viewed obliquely with respect to the synaptic ribbon. Here, the ribbon appears as a crescent with arciform densities at both ends. The red lines (also in C) highlight layers of dense material associated with the plasma membrane of the horizontal cells. Docked vesicles often faced these dense layers that are evocative of the post-synaptic densities in central synapses. The region inside the circle (arrow) represents the coated vesicle in the inset. Panel B shows a view of the rendered volume of the same reconstruction. The white dots indicate the location of vesicles that are hemi-fused with the plasma membrane (the docked pool). The inset shows the characteristic structure of these hemi-fused vesicles. The white circles indicate the tendency of these docked vesicles to be arranged in pairs. Panel C shows a single plane of a reconstruction where the synapse is viewed perpendicular with respect to the ribbon. Panel D shows a view of the rendered volume of the same reconstruction. As in B, the endings of horizontal cells are colored blue, docked vesicles are indicated by white dots and the white circles enclose pairs of docked vesicles. Vesicles with larger diameters than those of the synaptic vesicles were present in horizontal cell endings and bipolar cell dendrites (blue regions). Bar = 0.20 µm.

**Figure 3:**
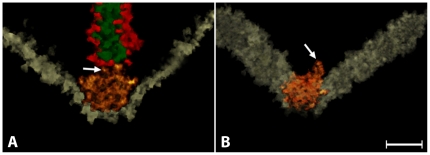
The arciform density. Panel A shows that the arciform density (orange) is shaped as a pentamer instead of an amorphous particle. Four sides of the pentagon are attached to the plasma membrane and the upper side to the plate of the ribbon via two short filaments (arrow). A central core (green) and surface densities for the attachment of the tethered vesicular pool (red) comprised the ribbon. The yellow line is the perpendicular view of the plane of the plasma membrane. Panel B shows a top view of the same arciform density. To gain a better view of this region, the ribbon was removed by density segmentation (see Materials and Methods). This top view reinforces the idea that the arciform density is a cage that links the ribbon to the plasma membrane (yellow). Bar: 50 nm.

The light-adapted rod photoreceptor axonal terminal or spherule (blue lines, Fig. 1A) measured 1.5±0.3 µm radius, 27±4 µm^2^ in surface area and 13±2 µm^3^ in volume (mean ± SD, n = 49; Table 2). The rod spherule contained a single ribbon (arrows, Fig. 1A) that was shaped as a crescent and measured 0.42±0.2 µm (mean ± SD, n = 49) in height and 0.030±0.003 µm (mean ± SD, n = 21) in width. The arciform density, a particle measuring ∼55 nm in diameter connected the ribbon's base to the plasma membrane (Figs. 2A–D; 3A–B). Other organelles in the cytoplasm included a prominent mitochondrion (M, Fig. 1A–B), a filamentous cytoskeleton matrix, synaptic vesicles (Fig. 1B reddish) and coated vesicles (inset, Fig. 2A). The plasma membrane surface area at the base of the ribbon measured ∼0.2 µm^2^. A synaptic triad made up of the rod terminal, horizontal cell axonal endings and bipolar cell dendrites was located at the base of the ribbon. An invagination containing the horizontal cell endings and bipolar cell dendrites in the rod spherule measured 8.7±1.5 µm^2^ in surface area and 2.5±0.5 µm^3^ in volume (mean ± SD, n = 49) (green lines, Fig. 1A).

Synaptic vesicles measuring ∼40 nm in diameter occupied ∼75% of the rod spherule volume in light-adapted mice retinas. In the tomograms, synaptic vesicles in the cytoplasm were spaced apart by 110–120 nm (center-to-center). Based on center-to-center spacing, we estimated between 580–750 synaptic vesicles per µm^3^ of cytoplasm or 5,800–7,500 synaptic vesicles per rod spherule. This is lower than an earlier estimate of 8,000–27,000 synaptic vesicles in the cytoplasm of a rod spherule [44]. We estimated in mouse rod spherules that 272–340 vesicles were tethered to the synaptic ribbon and 55–65 vesicles were located at the base of the ribbon. These values are also less than estimates of ∼640 to ∼770 synaptic vesicles tethered to the synaptic ribbon in salamander and mammalian rods, respectively [13], [20], [45] and the earlier estimate of ∼130 vesicles at the base of mammalian rod synaptic ribbons [45].

The analysis of the three-dimensional structure of the rod spherule identified a fourth pool, which we called “docked”. This pool contains 460–480 vesicles distributed in 3.0±0.5 µm^2^ (mean ± SD, n = 3) of active zone (∼150 vesicles/µm^2^). The vesicles of the “docked” pool were hemi-fused with the cytoplasmic leaflet of the plasma membrane (inset, Fig. 2B). Hemi-fused vesicles were located near the base of the synaptic ribbon as well as along the slope of the synaptic ridge, and in ribbon-free regions in apposition to the horizontal cell endings (white dots, Fig. 2B, D). Docked vesicles often faced the electron-dense plasma membrane specializations of horizontal cell endings that were associated with the base of the ribbon, and with plasma membrane regions located away from the ribbon, suggestive of post-synaptic densities of conventional synapses (red lines, Fig. 2A, C) [10], [46]. Thus, the docked vesicle pool was larger and occupied a greater area than the conventionally defined synaptic vesicle pool that is associated with the base of the ribbon and often identified as the readily releasable pool [45].

### Arciform density

We also studied the structure of the arciform density, which linked the base of the ribbon to the plasma membrane (Figs. 1B; 2A–D). Instead of an amorphous density as seen in conventional electron micrographs, our study showed the presence of particles shaped as cages measuring ∼55 nm in diameter and exhibiting pentagonal symmetry (Fig. 3A–B). In contrast to the two-dimensional grid arrangement in central synapses [9], the cages were arranged in columns along the base of the ribbon. Pairs of filaments rising from the upper surface connected the cage to the base of the ribbon (arrows, Fig. 3A–B). The position and structure of the filaments are consistent with their identity as Bassoon, a protein that is required for anchoring the ribbon via RIBEYE [47] to the arciform density [33]. Bassoon immunoreactivity is located at the base of the ribbon [48]–[50] and ribbons are distributed “free-floating” in the cytoplasm of photoreceptors in a *bassoon* null mouse mutant, which expresses a non-functional Bassoon protein [51], [52]. Furthermore, Bassoon immunoreactivity is diffusely distribution in the photoreceptor cytoplasm in the mutant, suggesting it is not an integral component of the ribbon [52].

### Light- and Dark-Adaptation

We studied the structural properties of rod spherules of mice placed in the dark for 3–5, 15, 30, 60 and 180 minutes (Table 2). We observed an increase in the dimensions of the horizontal cell axonal endings from 2.4±0.5 µm^3^ in light-adapted mice to 7.6±1.5 µm^3^ after 3–5 minutes and to 9.2±1.4 µm^3^ after 15 minutes in dark-adapted mice (column labeled Vol. HC, Table 2). The increase in the dimensions of the horizontal cell invaginations did not correspond to the changes of the rod spherule volume (blue lines, Fig. 1A; column labeled Vol. Terminal, Table 2), suggesting a reduction in the volume of the rod spherule cytoplasm.

**Figure 4:**
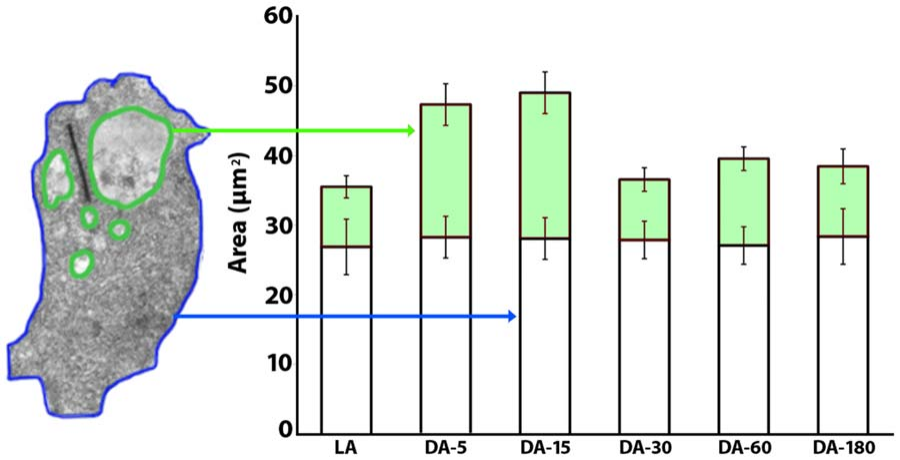
Changes in surface area. The histogram shows changes in the surface area of rod spherules and horizontal cell endings and bipolar cell dendrites (blue and green lines, left side) in the light (LA) and the dark (DA). The x-axis plots the time in minutes and the y-axis, the surface area in µm^2^. The surface area of the axon terminal did not change significantly (white rectangles). In contrast, the surface area of the horizontal and bipolar cell endings increased in mice left for 3–5 and 15 minutes in the dark (green rectangle). After 30 minutes in the dark, the horizontal endings returned to the same size as horizontal cell endings in the light, and they remained in the same size range up to 180 minutes in the dark.

We estimated the surface area of the region of membrane apposition between the rod spherule and the invaginating horizontal cell axonal endings (i.e., the region facing the rod spherule; green outlines, Figs. 1A–B & 4). We expected a small increase in the surface area of horizontal cell endings (∼0.18 µm^2^) to correspond to the estimated number of vesicles that fused with the plasma membrane based on the decrease in rod spherule cytoplasmic volume. However, the surface area of the invaginating horizontal cell axonal endings increased from 8.7±1.5 µm^2^ in light to 19±3 µm^2^ after 3–5 minutes and to 21.0±3 µm^2^ after 15 minutes in the dark (green, Fig. 4; Area HC endings; Table 2). After 30 minutes in the dark, the increase in the surface area of horizontal cell axonal endings reverted to the light-adapted condition and remained within this range for up to 180 minutes (Fig. 4).

**Figure 5:**
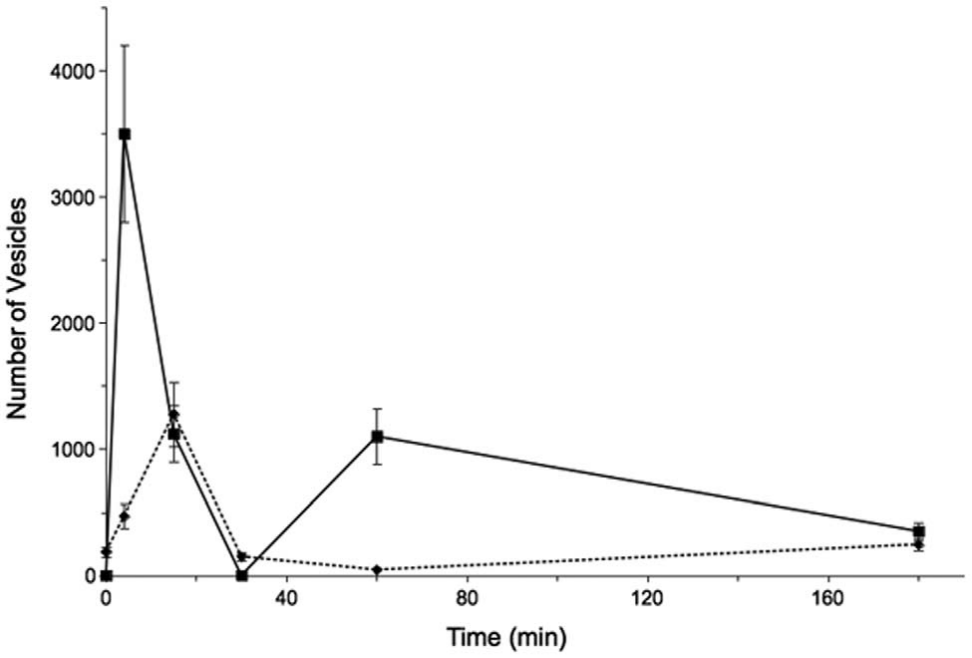
Vesicle fusion. The x-axis plots the time in the dark and y-axis plot the number of vesicles that fuse with the plasma membrane. The solid line indicates the number estimated from the decrease in the volume of the axon (mean ± SD). The dotted line indicates the number estimated from the increase in the number of omega figures (Table 2).

**Figure 6:**
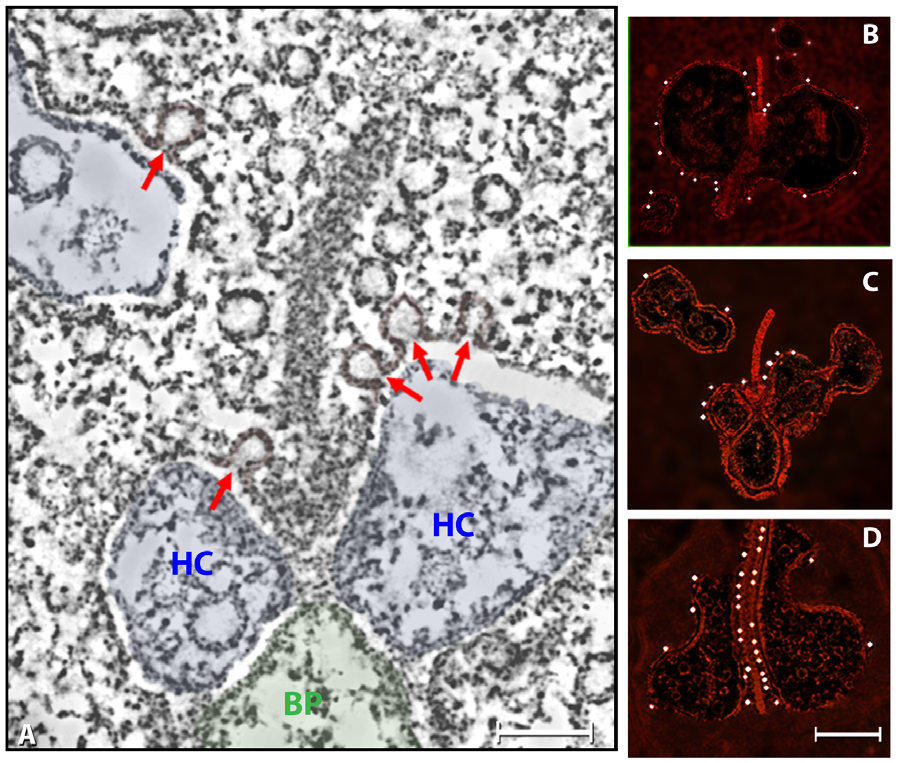
Distribution of fully fused vesicles or omega figures. Panel A shows a plane from the reconstruction of a ribbon synapse from a mouse dark-adapted for 15 minutes. The omega figures are located at the base of the ribbon and along the slope of the synaptic ridge, as well at ribbon-free sites. HC and BP indicate horizontal endings and bipolar cell dendrites, respectively. Panels B–D show the localization of omega figures (white dots) at ribbon-associated (panel B) and ribbon-free regions of the rod spherule (panels C–D). Bar: 60 nm.

We also estimated for the first time, the number of fully fused synaptic vesicles in rod spherules of light- and dark-adapted mice (Omega Figures/Terminal, Table 2; Figs. 5 and 6 A–D). The fully fused vesicles, based on their size and shape, and the lack of a cytoplasmic coat suggests they are exocytotic vesicles rather than endocytotic vesicles [53], [54]. The likelihood that the fully fused vesicles are exocytotic is also suggested by their increased number during the short periods of dark adaptation. There was an increase of fully fused vesicles from 187±34 per spherule in light-adapted animals to 470±55 at 3–5 minutes and 1,280±150 at 15 minutes in dark-adapted animals. Relative to the number of fully fused vesicles in the light condition (Table 2), the number of fully fused vesicles for these dark adaptation periods increased by ∼280% and ∼680%, respectively. The number of fully fused vesicles decreased to 475±65 per spherule at 30 minutes in the dark. The number of fully fused vesicles after 60 and 180 minutes in the dark was similar to the number of fully fused vesicles in the light condition.

We calculated that after 15 minutes in the dark, approximately one half of the vesicles in the rod spherule (2,900–3,750) would have fused with the plasma membrane (Fig. 4) based on the increase in the volume and the surface area of the invaginating horizontal cell axonal endings as well as the number of fully fused vesicles in the rod spherule (Table 2). After 30 minutes in the dark, the volume and the surface area of the horizontal cell axonal endings returned to values determined in the light-adapted condition (Vol. HC and Area HC endings, Table 2). The number of omega figures also returned to basal levels after 60 minutes of dark-adaptation. In addition, rod spherules from light-adapted retinas were indistinguishable from retinas dark-adapted for 60 or 180 minutes (Table 2).

Compared to estimates from changes in the volume of the rod spherule, however, the number of omega figures suggests that the number of vesicles fused at the active zone was smaller and that the peak number of omega figures occurred at 15 minutes of dark adaptation instead of 3–5 minutes of dark adaptation (Fig. 5).

Fully fused vesicles were distributed in a continuum that included both regions in the vicinity of the ribbon as well as at regions located away from the ribbon (Fig. 6A–D). In approximately 5% of the rod spherule reconstructions, most fully fused vesicles were clustered in ribbon-associated regions and a small number occurred in ribbon-free regions (Fig. 6B). However, in the majority of the reconstructions, the fully fused vesicles were distributed near the base of the ribbon, along the slope of the synaptic ridge and in ribbon-free regions (red arrows Fig. 6A and white dots in Fig. 6C, D). In most of the reconstructions, the majority of omega figures (∼60%) were associated with ribbon-free regions of the rod spherule in apposition to the horizontal cell axonal endings. Independent of their location in the active zone, fully fused vesicles exhibited the same diameter as the synaptic vesicles populating the cytoplasm or hemi-fused with the plasma membrane (Fig. 6A).

We also studied whether the number of hemi-fused vesicles that contact the plasma membrane changed during dark-adaptation. Having shown that the number of omega figures increased after 15 minutes in the dark (Omega Figures/Terminal, Table 2; Figs. 5, 6A), we expected that vesicles that are closest to the plasma membrane, and presumably the first to fuse with the plasma membrane following depolarization [55], [56] would decrease in number. In the dark, however, the number of hemi-fused vesicles per terminal was unchanged and fell within a narrow range (380–470). The sole exception occurred in mice exposed to the dark for 3–5 minutes where the number of hemi-fused vesicles increased by ∼30% (or ∼800) per terminal.

Finally, we evaluated the number of coated vesicles in rod spherules from mice in the light and dark conditions. We expected that the number of coated vesicles would increase with dark adaptation because photoreceptor transmitter release is continuous in the dark [36]. The increase in rod spherule plasma membrane area following vesicle fusion is presumably accompanied by an increase in the rate of compensatory endocytosis and the appearance of coated vesicles [53]. However, we found that the number of coated vesicles (35–40/terminal) in rod spherules from light-adapted retinas was similar to the number of coated vesicles in rod spherules from dark-adapted retinas at each time point.

## Discussion

**Figure 7:**
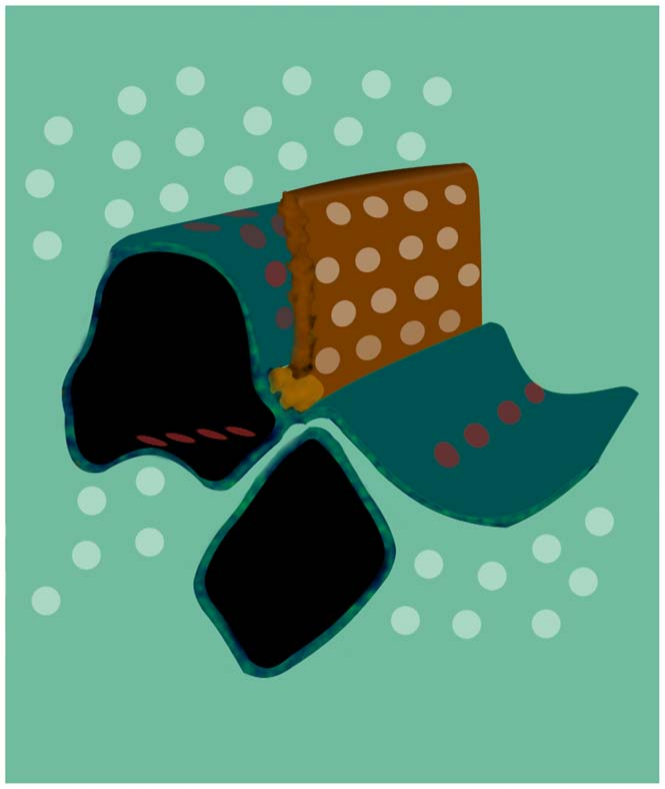
Model of rod photoreceptor ribbon synapse. To impart three-dimensionality, the synaptic ribbon, the tethered vesicular pool and the endings of the horizontal cells were extended along the z-axis. Docked vesicles are located at ribbon-associated (green) and at ribbon-free regions (red circles) the synaptic terminal. The distribution of docking and fusion sites along the entire synapse could account for the capacity of ribbon synapses to release glutamate in a rapid and sustained manner during prolong depolarization.

Our findings support a conventional mechanism for neurotransmitter release from rod photoreceptors where several thousand synaptic vesicles fuse at active zones in ribbon-associated and ribbon-free regions of the rod spherule that are in apposition to horizontal cell endings. Supporting this conclusion is a synchronized series of events that occur with depolarization of the rod spherule in the dark, including a decrease in the rod spherule volume (Table 2), a large increase of hemi-fused (docked) vesicles (Fig. 7) and an increase in the number of fully fused (omega figures) vesicles during the first 30 minutes of darkness (Table 2; Figs. 2A–D; 6A–D). There was an absence of intracellular cisterns or vacuoles near the synaptic ribbon that would have been indicative of compound vesicle fusion [57]–[60]. Finally, synaptic vesicles were not selectively depleted at the base of the synaptic ribbon [30] in the dark conditions used in these studies.

### Synaptic vesicle fusion at ribbon-associated regions

The synaptic ribbon, the prominent presynaptic structure in photoreceptor terminals, is associated with a large number of vesicles that are either attached to its surface by short filaments or are nearby the ribbon [11], [13], [14]. We defined the ribbon-associated region of the rod spherule by the presence of a synaptic ribbon, and included the plasma membrane at the base of the synaptic ribbon (synaptic ridge) and the adjacent plasma membrane [14]. There is a high density of hemi-fused and fully fused vesicles near the base of the synaptic ribbon and along the slope of the synaptic ridge in light and dark (Figs. 2B, D and 6B), consistent with earlier structural and physiological findings of transmitter release from this region of the photoreceptor terminal [18], [20], [22].

Several different functional roles have been proposed for synaptic ribbons, including participation in vesicle transport (i.e., conveyor belt, safety belt or capacitor), vesicle capture from the cytoplasm, vesicle priming for docking and fusion, and compound vesicle fusion [30], [45], [59], [61]–[64]. Synaptic ribbons could also play a role in clustering and organizing a high density of the molecular components that mediate synaptic vesicle docking and fusion [17]. The perpendicular orientation of the synaptic ribbon with respect to the plasma membrane allows for an increase in the number of hemi-fused or docked vesicles, since fusion sites occurs along both sides of the ribbon. Consistent with this idea is the localization near the base of the ribbon of several proteins that are associated with the cytomatrix of the active zone and participate in docking, priming and fusion of synaptic vesicles, including Munc13-1, RIM2 and CAST1 [33], [49], [65]–[68]. Furthermore, in central synapses, the presynaptic scaffolding protein, Bassoon participates in clustering of synaptic vesicles [69].

Voltage-gated Ca^2+^ channel subunits are also central components of the molecular matrix mediating vesicle exocytosis at synaptic ribbon active zones. An earlier freeze fracture study described large intramembrane particles aligned with the base of the ribbon [14]. These likely include the voltage-gated Ca^2+^ channels [70], [71]. There is also a higher level of α_1_ subunit Ca^2+^ channel immunostaining near the base of the synaptic ribbon [33], [72], [73]. These observations are consistent with a higher density of Ca^2+^ entry sites near synaptic ribbons or bodies in cochlear inner hair cells, bipolar cells and cone photoreceptors [22], [25], [26], [31], [74]–[77].

Furthermore, mouse mutants lacking either the β_2_ or Ca(v) 1.4 α_1_-Ca^2+^ channel subunits are characterized by an abnormal or absent ERG b-wave, and for the Ca(v) 1.4 α_1_-Ca^2+^ channel subunit mutant, there is a loss of synaptic ribbons in the rod spherules [78]–[81]. A mouse mutant lacking CaBP4, a calmodulin-like Ca^2+^ binding protein that interacts with Ca(v) 1.4 Ca^2+^ channels, also shows defects in the ERG b-wave and reduction of the number of synaptic ribbons [82]. Findings from these mutants are congruent with the idea that Ca^2+^ channels in photoreceptors are closely associated with the synaptic ribbon.

The findings of a high density of hemi- and fully fused synaptic vesicles (Fig. 6), and the molecular components, including Ca^2+^ channels that participate in transmitter release at ribbon-associated regions of the rod spherule suggests a highly efficient positioning of docked vesicles in the ribbon active zone. This arrangement would support rapid transmitter release from rods including synchronous fusion of multiple vesicles that might occur with strong depolarization [37], [58], [60], [83]–[86].

### Synaptic vesicle fusion at ribbon-free regions

Hemi-fused and fully fused vesicles were commonly observed in ribbon-free regions of rod spherules in apposition to horizontal cell axonal endings in both light- and dark-adapted retinas (Fig. 6). These findings extend earlier reports of docked vesicles and clusters of vesicles in ribbon-free regions of goldfish bipolar cells following treatment with Co^2+^ and the PKC activator phorbol 12-myristate [24], [25]. Furthermore, extensive depletion of docked vesicles by strong K^+^ depolarization from regions up to 800 nm from the synaptic body of saccular hair cells [23] supports the idea that vesicle fusion occurs at active zones in ribbon-free regions of the rod spherule.

Increasing the Ca^2+^ levels in rod spherules to mediate vesicle fusion in ribbon-free sites could be accomplished by several different mechanisms. For instance, Ca^2+^-induced Ca^2+^ release from endoplasmic reticulum, which elevates intracellular Ca^2+^ levels in rod spherules and enhances sustained transmitter release from salamander and mouse rods [87]–[90], could evoke vesicle fusion. In addition, there could be an increase of intracellular Ca^2+^ by diffusion from Ca^2+^ entry sites at or near the synaptic ribbon as suggested for goldfish bipolar and hair cells [25], [76], [91]. Focal synaptic vesicle fusion at ribbon-free sites may be evoked directly by Ca^2+^ from local Ca^2+^ channels, since diffuse L-type Ca^2+^ channel immunostaining is distributed to all regions of the salamander [92]–[94] and mouse rod spherule [72], [73]. Increases of rod spherule Ca^2+^ levels would support both evoked and spontaneous synaptic vesicle exocytosis at ribbon-free sites of bipolar cell terminals and hair cells [22], [24]–[26], [95].

Reduced and altered photoreceptor to bipolar cell synaptic transmission occurs in *bassoon* mutants, which are deficient in a functional Bassoon protein [52]. In rod photoreceptors of the *bassoon* mutant, neurotransmission is thought to be mediated by ectopic ribbon synapses and a few remaining ribbon synapses [52]. However, synaptic vesicle fusion could also occur at active zones in ribbon-free sites of *bassoon* mutant rod spherules, since postsynaptic elements (fig. 3; [52]) and several presynaptic proteins that participate in synaptic vesicle docking and fusion remain in the mutant rod spherules [33]. Furthermore, pan α1 and Ca(v)1.4 α1 Ca^2+^ channel immunoreactivity are diffusely distributed in the terminals of young *bassoon* mutants [73]. Hemi-fused and fully fused synaptic vesicles observed at active zones in ribbon-free regions of wild type mouse rod spherules (Figs. 6 & 7), if present in the *bassoon* mutant, would likely support neurotransmission. Consistent with this idea is the slow and sustained exocytosis from *bassoon* mutant cochlear inner hair cells, which lack anchored ribbons, but have docked synaptic vesicles [84], [96].

### Omega figures

Fully fused vesicles at the plasma membrane are commonly referred to as “omega figures”. The membranes of fully fused vesicles are continuous with the plasma membrane and they form a pore between the extracellular space and the lumen of the vesicle. Omega figures and synaptic vesicles have a similar size and they lack a cytoplasmic coat in electron micrographs [1], [53], [97]. Their presence is inferred to reflect vesicle exocytosis and transmitter release based on classic studies of the neuromuscular junction [98].

Omega figures were readily apparent in the rod spherule tomograms, and they had a similar appearance in *all regions* of the rod spherule (Fig. 6A–D). They were similar in size to vesicles in the cytoplasm and tethered to the synaptic ribbon. Furthermore, there were no obvious differences in their cytoplasmic coats. Together these findings support our proposal that the omega figures reflect exocytosis in both ribbon-associated and ribbon-free regions. An alternative hypothesis, that omega figures at the ribbon mediate exocytosis and omega figures at ribbon-free regions mediate endocytosis, suggests different functional roles for identically appearing organelles, an unlikely and unprecedented possibility. The omega figures differed markedly in their appearance from coated pits and other endocytotic intermediates involving clathrin that were readily visualized in the tomograms (not shown) [53], [54], [99]. The presence of coated pits and vesicles, a feature of clathrin-mediated mechanisms, is reported in isolated photoreceptors, and in photoreceptor terminals *in situ* and *in vitro* (retinal slices) [11], [100]–[105]. Consistent with these observations is a high level of expression of clathrin and clathrin accessory proteins, including dynamin and amphiphysin in photoreceptor terminals [106], [107]. Furthermore, the omega figures are not likely to be synaptic vesicles undergoing a “kiss-and-run” exocytosis, where synaptic vesicles have a transient fusion with the plasma membrane [108], [109]. This mode of exocytosis is not present or rare at conventional synapses [110]–[112]. “Kiss-and-run” exocytosis has not been observed in bipolar cells [24], [26], [113] and it is unlikely to occur in photoreceptors [44]. Therefore, the most parsimonious explanation for the omega figures observed in both ribbon-associated and ribbon-free regions are fused synaptic vesicles undergoing exocytosis.

### Exocytosis and endocytosis; changes in the rod terminal surface area and volume

To evaluate vesicle exocytosis and endocytosis in the rod spherules, we measured simultaneously the *surface area and the volume* of the invaginating horizontal cell endings in light- and dark-adapted mice (Table 2) and compared these changes to the number of fully fused vesicles. Mice dark-adapted for 3 to 15 minutes showed an *increase* in the surface area of the contact between the rod spherule and horizontal cell endings, which correlated with an increase in the number of fully fused vesicles, and a *decrease* in the volume of the rod spherule. Based on these findings, we conclude that vesicle exocytosis a) accounts principally for the increase in the number of fully fused vesicles, and b) contributes to the synchronized expansion of the rod terminal surface area and the decrease in the volume of the rod spherule during the short duration dark conditions. Therefore, in mice dark-adapted for 30 minutes or more, the *decrease* in the surface area of the axon spherule in apposition to horizontal cell endings (Table 2) is due to compensatory endocytosis.

The decrease in the volume of the cytoplasm of rod spherules suggests that 2,500–3,000 synaptic vesicles fuse at rod spherule active zones during the first 30 minutes of dark-adaptation. On this basis, we expected a modest increase of the surface area of the rod synaptic contact reflecting vesicle fusion at the active zone. Unanticipated however, was the marked increase in rod terminal surface area, which would require >200,000 vesicles fusing with the plasma membrane to compensate for the loss in the spherule's volume. Since the rod spherule cytoplasm is estimated to contain 5,800–7,500 synaptic vesicles, we propose that a mechanism opposing the loss of the rod spherule volume involves both an increase in rod terminal surface area concomitant with swelling of the horizontal cell endings (a balloon-within-a-balloon). A consequence of this “balloon-within-a-balloon” model is the expectation that horizontal cell endings are dynamic and prevent the collapse of rod spherules by swelling and shrinking during early stages of dark-adaptation.

Morphological changes similar to those described by a “balloon-within-a-balloon” model have been observed in other vertebrate photoreceptors. Photoreceptor membrane invaginations called diverticula are located in rod spherules [13], [100], [114], [115]. In cat rod spherules, there was a slight increase of the surface area (0.6±0.56 vs. 1.42±0.61 µm; p<0.1) of diverticula at two hours of dark-adaptation [13]. Interestingly, an earlier qualitative study of the chicken retina reports extensive invaginations within the rod spherule that are greatest an hour after the onset of darkness and their disappearance after three hours in the dark [100]. Coated vesicles, which are observed in both the mouse and chick retina, appear to be more frequent in the chick retina, and this may be related to the extent of the membrane invaginations and the longer periods of dark adaptation used in this study. Together, these observations suggest that the rates of exocytosis and endocytosis in rod spherules differ at the beginning of the dark-adaptation period in both mouse and chicken, and that these rates equilibrate over a period of 30 minutes for mouse (Table 2) and 180 minutes for chicken rod spherules. The similarity of the time course in the changes of spherule area in these two species, suggests the same cellular mechanisms mediate exocytosis and endocytosis during dark-adaptation in vertebrate rod photoreceptors.

There are other dynamic changes in the structure of non-mammalian photoreceptor terminals in light and dark [103], [116]. For instance, in turtle cones, after one-hour in the dark, there is extensive invagination of the pedicle that is reversed by light exposure; in darkness, there is an increase in the surface density of the invaginating processes, consistent with our observations of an increase in the surface area of the rod spherule at the beginning of dark-adaptation. There are also dynamic morphological changes of horizontal cell endings associated with synaptic activity in teleost cones during light adaptation [116]–[118]. Fish horizontal cell dendritic spinules appear and increase the synaptic contact area in light-adapted cone terminals and they rapidly disappear at light offset. Spinule dynamics are concurrent with the changes in the feedback signal recorded from horizontal cells, suggesting they are sites of horizontal cell-cone communication, and that changes of the area of synaptic contact in fish cone synapses could provide a mechanism for cone modulation [116], [119]. These findings with our observations of changes in rod spherule size with dark adaptation are consistent with a high level of structural plasticity of photoreceptor synapses.

### Exocytosis; docked vesicle pool

We anticipated that the hemi-fused (docked) vesicle pool would decrease in rod spherules during dark adaptation, based on an earlier study in which, following depolarization with high-K^+^ saline, saccular hair cell ribbon synapses showed a decrease of 73% of the number of docked vesicles at ribbon-associated and ribbon-free zones [23]. Similarly, strong depolarization of goldfish bipolar cells results in a loss of about 50% of the synaptic vesicles tethered to the synaptic ribbon [59]. Surprisingly, in the rod spherule, we found that the number of docked vesicles was independent of the length of time that the mice were left in the dark. The differences between our findings and the saccular hair cell and bipolar cell studies may be due to differences in the experimental design. Whereas we used an *in situ* preparation with vesicle release evoked by darkness, the *in vitro* preparations with the saccular hair cells were depolarized by high K^+^ for 30 minutes and the bipolar cells were depolarized by strong electrophysiological stimulation or high K^+^. The strong depolarization protocols used for the hair cell and the bipolar cell presumably evoke greater vesicle fusion than the dark evoked depolarization of rods.

In summary, shortly after dark onset, depolarization induces several thousand synaptic vesicles to fuse at active zones in rod spherules. The distribution of hemi-fused and fully fused vesicles indicates that docking and exocytosis occurs along the entire region of the rod spherule facing the horizontal cell axonal endings, and not just at or near the synaptic ribbon's base (Figs. 6 & 7). The distribution of vesicle fusion sites supports the hypothesis that the entire pre-synaptic terminal, not just the limited region around the synaptic ribbon's base, participates in transmitter release from rod photoreceptors.

## Materials and Methods

### Ethics Statement

Animal care and use protocols (ARC #1998-064-41A and ARC #1998-014-13C) were approved by the UCLA Animal Care and Use Committee. All of the animal studies were performed in accordance with ARVO's Use of Animals in Ophthalmic and Visual Research and PHS Policy on Humane Care and Use of Laboratory Animals. Male and female C57Bl/6J mice, 10 to 12 weeks old (The Jackson Laboratories, Bar Harbor, Maine), were housed with 12 hour light-dark cycle. Light-adapted mice were housed under room illumination (∼750 lux) and the eyes were collected at the mid-point of the light phase. Dark-adapted mice were placed into a light-tight box at the mid-point of the light phase for 3–5, 15, 30, 60 and 180 minutes. Mice were euthanized by cervical dislocation, which is approved by the AVMA Panel on Euthanasia (2000).

### Preparation of Specimens

The eyes were rapidly removed and dissected under a dim red light (Wratten IR filter 87C). The eyecups were washed briefly in HEPES/NaCl/glucose buffer, pH 7.2 and immersed in a solution containing 2.5% paraformaldehyde and 2% glutaraldehyde in 0.1 M Na phosphate buffer, pH 7.2, for 3 hours at room temperature. Dissection of the eye and fixation of the eyecup was estimated to take 1–3 minutes.

The eyecups were cut into quadrants and immersed in 0.1 M Na cacodylic buffer, pH 7.4, with 3% glutaraldehyde and 0.5% tannic acid for 30 minutes at room temperature. They were washed in 0.1M Na cacodylic buffer and 4% sucrose, post-fixed in OsO_4_ for 90 minutes and incubated in 0.5% uranyl acetate for 48 hours at 4°C. The retinal samples were dehydrated in graded solutions of ethanol and passed through one change of propylene oxide. The quadrants were trimmed to 2 mm^2^ pieces, embedded in Epon and cured for 48 hours at 60°C. Thin sections (grey-to-silver interference color) were cut perpendicular to the vitreous using a *RMC MTX* Ultramicrotome. Sections were collected on carbon-coated mesh grids and stained with uranyl acetate and lead citrate. For all conical series, 10 nm diameter gold particles, used as fiduciary markers for image alignment were deposited on the sections before coating them with carbon.

### Conical Tomography

All methods were developed in our laboratories and have been described previously [9], [38]–[43]. For each experimental condition (Table 1), we collected three conical series using a Gatan 650 Single Tilt Rotating Holder in a FEI Tecnai 12 electron microscope at 120 KV. The images were collected with a 2k×2k CCD Gatan camera using a minimum-dose method: searching the section at a magnification of 2,700× and focusing away from the area of interest. The specimens were tilted at 55° and rotated in 5° increments through a complete 360° turn. Collection of the conical series started with an un-tilted projection of the region. After completing the series, the specimen was brought back to 0° tilt to collect a final projection. The difference in the distance between the same gold particles in both projections was used to determine specimen shrinkage from radiation damage.

**Table 1:** List of reconstructions.

Reconstruction	Condition	Magnification
05_28	Light	11,000
06_03	Light	11,000
07_10	Light	11,000
06_26	Dark (3–5 min)	11,000
06_30	Dark (3–5 min)	11,000
08_03	Dark (3–5 min)	11,000
05_11	Dark (15 min)	15,000
05-13	Dark (15 min)	15,000
05_22	Dark (15 min)	15,000
04_22	Dark (30 min)	11,000
05_05	Dark (30 min)	15,000
05_19	Dark (30 min)	15,000
07_29	Dark (60 min)	11,000
07_31	Dark (60 min)	11,000
08_01	Dark (60 min)	11,000
06_12	Dark (180 min)	11,000
06_15	Dark (180 min)	11,000
08_04	Dark (180 min)	11,000

**Table 2:** Changes in rod spherules during dark adaptation.

	Vol. Terminal (µm^3^)	Vol. HC Endings (µm^3^)	Area HC Endings (µm^2^)	Omega Figures/Terminal
**Light-adapted**				
	13.0±2.0 (49)	2.4±0.5 (49)	8.7±1.5 (49)	187±34 (3)
**Dark-adapted**				
3–5 min	14.0±1.5 (32)	7.6±1.5 (32)	19.0±3.0 (32)	470±55 (3)
15 min	13.8±1.5 (28)	9.2±1.4 (28)	21.0±3.0 (28)	1280±150 (3)
30 min	13.5±1.5 (33)	2.4±1.0 (33)	9.0±1.0 (33)	475±65 (3)
60 min	13.0±1.5 (46)	4.2±1.0 (46)	12.5±3.0 (46)	45±9 (3)
180 min	14.0±2.0 (41)	3.0±1.0 (41)	10.0±3.0 (41)	246±32 (3)

The measurements express mean±SD and the number of measurements in parentheses. The number of omega figures was the average contained in three reconstructions.

Vol. = volume; HC = horizontal cell.

Alignment and preliminary reconstruction required putting the three Euler angles, α, β, and γ, and the x and y shifts into a common reference system. The x and y shifts were obtained from the coordinates of the gold particles that were deposited on the surface of the section. First, a gold particle was selected as the center for all the projections of the conical series. After centering, the images were aligned using the coordinates of 5–6 gold particles present in all images of the series. After alignment, preliminary three-dimensional maps were calculated using a weighted back projection algorithm. To improve resolution, the preliminary maps were refined using projection matching. First, we performed a global alignment where the projections were iteratively cross-correlated with re-projections of an updating reconstruction. Second, a local alignment was performed to correct for the deformations induced by radiation damage. This strategy involved: a) partition of the preliminary reconstruction into sub-volumes, b) extraction of corresponding sub-areas for each sub-volume from the micrographs of the tilt series, c) re-projection of each sub-volume according to the orientation parameters, and d) refinement of these parameters by correlating each sub-area to the corresponding computed projection. The thickness of the plasma membrane was also used to estimate the resolution of the refined conical maps. Since there was a significant variability in the distribution of the densities comprising the triple-layered unit membrane structure, we measured the distance between the centers of the layers in the plasma and vesicular membrane. Consistent with our work on neocortical synapses [9], [41]–[43], the resolution of the conical maps at 11,000× was ∼6 nm, and at 31,000× it was ∼3 nm in all directions.

The *Amira* (www.amiravis.com) software package was used to visualize the reconstructed synapses as well as those segmented using *JUST* (Java User Segmentation Tool), a program that combines the 3D watershed algorithm with supervised classification [40]. Semiautomatic segmentation involves: a) creation of a three-dimensional watershed map of the volume, b) extraction of the background noise, c) extraction of vesicles, plasma membranes and gold particles, d) extraction of regions with high, medium and low densities and e) composition of a final segmentation map where all segmented structures were analyzed and conflicting assignments resolved.

Measurements of the volume and the surface area of both the rod spherule and the horizontal cell endings cells were estimated from single projections using the *ImageJ* software package (rsbweb.nih.gov/ij/). For each experimental condition, we traced the outline of the rod spherule, horizontal axonal endings and bipolar cell dendrite using the freehand selection tool (green, Fig. 1A). From the perimeters, the radius of the equivalent sphere was calculated and used to estimate surface areas and volumes. Since the bipolar dendrites are smaller and did not change like the horizontal cell endings, they were included in the calculation.

The number of hemi- (docked) and fully fused (omega figures) vesicles was obtained from the conical tomograms. To estimate surface area, we measured the length of the membrane in the x-y plane and counted the number of slices that contained the membrane in the z-plane. Next, we estimated the number of docked vesicles and omega figures. Vesicles where the region of contact was a single leaflet of the plasma membrane represented the docked pool (Inset, Fig. 2A). Small, uncoated vesicles that are fused with the plasma membrane and form a pore that is continuous with the extra-cellular space were defined as omega figures. Since the reconstruction was ∼50 nm in thickness, hemi-fused vesicles and omega figures with more than 2/3 of the diameter within the reconstructed volume were counted as 1, those which were one half of their diameter as one-half and less than that were not included in the counts.

The number and distribution of hemi- and fully fused vesicles relative to the plasma membrane was determined by labeling their centers using the landmark editor feature of *Amira* (Figs. 2 B, D; 6 B–D). The landmark function was also used to measure the center-to-center spacing of vesicles at the plasma membrane, synaptic ribbon and distributed to in the cytoplasm. The distance between neighboring vesicles was calculated using the formula: √((x_0_−x_1_)^2^+(y_0_−y_1_)^2^+(z_0_−z_1_)^2^), where x, y and z are the coordinates of these centers. The angle formed by three consecutive vesicles was calculated with the formula: arccos((v1•v2)/(|v1|*|v2|)), where v1 and v2 are vectors, (•) is the dot product and |v1|*|v2| are the multiplied normalized vectors. The thickness of the membranes was estimated from the distance between the centers of the dense layers flanking the electron-lucent core [9], [42], [43].
